# Effect of climate on incidence of respiratory syncytial virus infections in a refugee camp in Kenya: A non-Gaussian time-series analysis

**DOI:** 10.1371/journal.pone.0178323

**Published:** 2017-06-01

**Authors:** Raymond Nyoka, Jimmy Omony, Samuel M. Mwalili, Thomas N. O. Achia, Anthony Gichangi, Henry Mwambi

**Affiliations:** 1 School of Mathematics, Statistics and Computer Science, University of KwaZulu- Natal, Scottsville, South Africa; 2 Molecular Genetics Department, University of Groningen, Groningen, Netherlands; 3 Statistics Department, Jomo Kenyatta University of Agriculture and Technology, Nairobi, Kenya; 4 Jhpiego - an affiliate of John Hopkins University, Westlands, Nairobi, Kenya; Kliniken der Stadt Köln gGmbH, GERMANY

## Abstract

Respiratory syncytial virus (RSV) is one of the major causes of acute lower respiratory tract infections (ALRTI) in children. Children younger than 1 year are the most susceptible to RSV infection. RSV infections occur seasonally in temperate climate regions. Based on RSV surveillance and climatic data, we developed statistical models that were assessed and compared to predict the relationship between weather and RSV incidence among refugee children younger than 5 years in Dadaab refugee camp in Kenya. Most time-series analyses rely on the assumption of Gaussian-distributed data. However, surveillance data often do not have a Gaussian distribution. We used a generalized linear model (GLM) with a sinusoidal component over time to account for seasonal variation and extended it to a generalized additive model (GAM) with smoothing cubic splines. Climatic factors were included as covariates in the models before and after timescale decompositions, and the results were compared. Models with decomposed covariates fit RSV incidence data better than those without. The Poisson GAM with decomposed covariates of climatic factors fit the data well and had a higher explanatory and predictive power than GLM. The best model predicted the relationship between atmospheric conditions and RSV infection incidence among children younger than 5 years. This knowledge helps public health officials to prepare for, and respond more effectively to increasing RSV incidence in low-resource regions or communities.

## Introduction

Respiratory syncytial virus (RSV) is one of the major causes of acute lower respiratory tract infections (ALTRI) in infants and young children [1][2]. RSV infections occur seasonally in temperate climate regions [3]. RSV adversely impacts the health of adults and immunocompromised patients, and is associated with significant mortality and morbidity, particularly in young children and vulnerable infants [4]. Children younger than 1 year are most susceptible to RSV infection; often 60–70% of children in this age group have been infected at least once, and re-infection can occur throughout their lifetime [4][5][6].

RSV is shed in saliva and nasopharyngeal secretions [7]. Infected hosts shed higher quantities of viral particles upon exposure to higher-ambient temperatures [8]. Low humidity during winter enhances RSV viability, and enables its survival for up to 12 hours on nonporous surfaces [9]. In dry air conditions, large droplets evaporate and remain air-borne for longer periods of time. Some studies have shown that airborne transmission appears to be sensitive to ambient humidity and temperature in temperate regions [8][10]. RSV outbreaks show some seasonality that suggests a connection with atmospheric and environmental conditions [11][12]. Most RSV infections in temperate locations occur between November and April [13]. RSV infection has been associated with winter in these regions because people spend more time indoors, potentially in crowded conditions [14]. Such climatic regions are different from those of Kenya, which is located on the equator and experiences bimodal seasonal rainfall due to the interaction of the Northern and Southern Hemisphere monsoon systems [15]. However, variations in climatic factors, such as humidity, temperature, wind speed, rainfall etc., can have a significant impact on disease dynamics. Therefore, it is essential that the RSV incidence be evaluated for equatorial climatic regions to aid accurate predictions of RSV outbreaks. [16][17].

The wide range of statistical methods used to explore the link between RSV outbreaks and climate makes it difficult to elucidate a definitive relationship. Pearson correlation analysis was previously used to explain the associations of RSV-positive cases with meteorological variables [11]. The univariate analysis of variance (ANOVA), multiple regression analysis, and Spearman’s rank correlation were used to assess the association between RSV incidence and meteorological parameters [18]. A better understanding of the relationship between climate and RSV helps in making reliable predictions of its incidence.

Worldwide, as of 2005, 99% of deaths from RSV were reported by the World Health Organization (WHO) to occur in developing countries [19]. It is, therefore, crucial to establish good RSV surveillance systems in developing countries to help understand the dynamics of the disease. In 2006, the U.S. Centers for Disease Control and Prevention (CDC) and the Kenya Medical Research Institute (KEMRI) established a respiratory illness surveillance system to detect disease outbreaks in Kenyan refugee camps [20]. We used RSV incidence data from this system to explore the best model that predicts the relationship between RSV incidence and climatic factors along spatio-temporal scales to determine whether a seasonal pattern of RSV infection exists. A generalized linear model (GLM) with a sinusoidal component over time was used to account for seasonal variation and compared with a generalized additive model (GAM) with smoothing cubic splines. Climatic factors were included as covariates in the models before and after timescale decompositions.

## Methods

### Data

Surveillance for viral respiratory illnesses, including adenovirus, human metapneumovirus, influenza virus, parainfluenza viruses 1, 2, and 3, and RSV was implemented in Dadaab refugee camp in north eastern Kenya in 2007. Paediatric and adult patients who presented at a camp medical unit, and met the case definition for influenza-like illness (ILI) or severe acute respiratory infection (SARI), were enrolled into the laboratory-enhanced respiratory surveillance system and tested for all of the above diseases after an informed consent form was completed by adults, older minors, and guardians of all minors <15 years [20]. The number of laboratory-confirmed cases was recorded on a daily basis from September 2007 to August 2011. The monthly counts of all RSV cases among children younger than 5 years were included in the present analysis; the main outcome of interest being monthly RSV incidence rate in this age group. RSV incidence rate per 1,000 children younger than 5 years was calculated by dividing monthly RSV counts by the monthly population of children younger than age 5 years in the camp. Local weather and climatic data, including: the mean temperature and mean dew point for the day (both in °F); mean sea level pressure for the day in millibars; mean visibility for the day in miles; mean wind speed for the day in knots; minimum and maximum temperature (°F) reported during the day; and the total precipitation (in inches) reported during the day were obtained from the World Meteorological Organization’s (WMO’s), World Weather Watch Program, according to WMO Resolution 40 (Cg-XII) (available at http://www7.ncdc.noaa.gov/CDO/cdo). The meteorological dataset consisted of measurements recorded at successive, equally spaced time points (covariates used in the present study are provided in the supplementary materials, S3 Table). Data and R codes used in the analysis are available at https://figshare.com/s/feb61d236cad0abcf5b6 DOI 10.6084/m9.figshare.5010767.

### Statistical modeling

A Poisson distribution model was used in this analysis, as the outcome of interest (incident RSV cases) was non-Gaussian count data. Some authors have used Gaussian vector autoregressive models on multivariate counts that are serially correlated. Brandt and others used vector autoregressive methods that were based on Gaussian error process [21]. However, such an assumption is not applicable to event count data because it produces biased estimates [22]. So, as many of those methods apply for count series that approximate normality, they may not hold to dynamic events like the ones applied here. In the first model, seasonal effects on RSV incidence were analysed by using a generalized linear model (GLM) with a sinusoidal component to account for seasonal variation. The second model extended the GLM model to a generalized additive model (GAM) by applying smoothing cubic splines. The GAM is an extension of the GLM and is adaptable to non-normally distributed variables [23]. GLM uses linear predictors specified as the expected value of a response variable (*Y*_*j*_), which is expressed as *η* = *Σ*_*j*_*β*_*j*_(*X*_*j*_). Here, *β*_*j*_ is a coefficient parameter and *X*_*j*_ represents the *j*-th explanatory variable. The GAMs extend these by replacing them with *η* = *Σ*_*j*_*f*_*j*_(*X*_*j*_), where *f*_*j*_(*X*_*j*_) are unspecified nonparametric functions estimated by including smoothing splines [24]. GAMs allow for adjustments of the nonparametric, nonlinear, confounding effects of seasonality, trends, and weather variables, which have been previously used in modeling time-series data [25]. In the present analysis, climatic time-series covariates were included in the GLM and GAM models and implemented in R language v3.1.0 [26]. Both models were optimized for predictive accuracy and precision.

Data were decomposed into three components, namely: trend, seasonal, and random components, in order to independently evaluate the existence and strength of associations between RSV incidence and covariates on each time scale. Data decomposition was accomplished using Loess smoothing, a regression method that assigns a weighted polynomial to each component [25]. We introduced a GLM for time-series data, with a sinusoidal component over time to account for seasonal variations. The GLM was extended to include a smoothing function using the GAM approach to the Poisson distribution [27] In each model, a data-driven smoothing function of time was fitted, and compared with those fitted, using sine and cosine functions in the Fourier basis.

The observed number of RSV counts, *Y*_*t*_ at a given month *t* = 1, ⋯, *n* from the population at risk is assumed to follow a Poisson random variable: *Y*_*t*_ ∼ Poisson(*μ*_*t*_). We let *n*_*t*_ be the population of children age 5 years and younger at risk at time *t* in the camp. The expected value of *Y*_*t*_ is *E*(*Y*_*t*_) = *μ*_*t*_ = *n*_*t*_Ɵ_*t*_ where the dependence of covariates on Ɵ_*t*_ is modeled by ${\text{Ɵ}}_{t}=e^{x_{t}^{T}\beta }$. Therefore, a Poisson GLM of the form $E(Y_{t})=\mu_{t}=n_{t}e^{x_{t}^{T}\beta }$ is used. More explicitly, to model the incidence, we use:

$$\text{log}\mu_{t}=\beta_{0}+\alpha y_{t-1}+\sum_{t=1}^{n}\sum_{k=1}^{m}\sum_{s=1}^{r}\sum_{l=0}^{q}\beta_{ksl}x_{\left(t-l\right)ks}+\eta_{1}cos\left[\frac{2\pi t}{T}\right]+\eta_{2}sin\left[\frac{2\pi t}{T}\right]+\text{log}n_{t}$$ (1)

Where μ_*t*_ is the infection rate for the month, *t*. *β*_0_ is the intercept, *α* is the coefficient of the lagged RSV counts by one month, which is represented by *y*_*t*−1_, *x*_(*t*−*l*)*ks*_, is the decomposed measured covariate, *β*_*ksl*_ their corresponding coefficients with *k* = 1, ⋯, *m* covariates and *s* = 1, ⋯, *r* corresponding to *r*-th decomposition of the *k*-th covariate, *l* = 0, 1, ⋯, *q* distributed lags where *q* is the maximum lag and *t* = 1, ⋯, *n* are the time points. The terms *η*_1_ and *η*_2_ are coefficients of the sine and cosine function, respectively. Here, *T* is the number of time periods described by one cosine function over the interval [0,2*π*].

Using a cosine function, we specified two periods: one that defines the measure of RSV infection (month) and the other that is described by one cosine cycle. After fitting all covariates in the GLM model, the most parsimonious model was identified. The maximum lag for each covariate was obtained by comparing different lagged models using Akaike information criterion (AIC). The maximum lag for each covariate was used to run “crossbasis” in the “dlnm” package for time-series models [28][29]. The same covariates were used to fit the GAM model.

The corresponding GAM for the Poisson model is:

$$\text{log}\mu_{t}=\beta_{0}+\alpha y_{t-1}+\sum_{t=1}^{n}\sum_{k=1}^{m}\sum_{s=1}^{r}\sum_{l=0}^{q}\beta_{ksl}\Psi_{k}(x_{\left(t-l\right)ks},\lambda_{tks})+\Psi_{k+1}\left(t,\lambda_{t\left(k+1\right)}\right)+\text{log}n_{t}$$ (2)

Where *λ*_*tks*_ is the smoothing parameter or the degrees of freedom for covariates, *λ*_*k+*1_ is a smoothing parameter for time and Ψ. is the smoothing function. Larger values of *λ*. are indicative of a less-smooth function.

For Models (1) and (2), the additive time-scale decomposition of the *k*-th covariate into the seasonal (S), trend (T), and random (R) components is:

$$\beta_{ksl}x_{\left(t-l\right)ks}=\beta_{kSl}x_{\left(t-l\right)kS}+\beta_{kTl}x_{\left(t-l\right)kT}+\xi_{\left(t-l\right)kR}$$ (3)

for every *k* in {1, ⋯, *m*}. In the above case, *s* takes on three levels S, T, and R. This decomposition helps in assessing for the significance of the seasonal and trend components of the covariates in explaining the RSV incidence. The combination of the seasonal and trend components makes up the patterns in the covariates.

The trend cycles represent long-term changes in the levels or values of the covariate, while the periodic changes are the fluctuations of constant length. The GLM (1) has the Logit link function. The residual deviance for these models takes on the form *D* = −2log(*L*_*test*_/*L*_*sat*_), where *L*_*test*_ and *L*_*sat*_ are the maximized likelihoods under the test and saturated models, respectively. The model selection and fitting was done using the "glmulti" package [30] and "gam" [31] in "mgcv" package [24] in the R language v3.1.0.

### Ethical considerations

Ethical approval for the surveillance activities was obtained from the KEMRI Ethical Review Committee (SSC Protocol Number 1161). Institutional review was waived by CDC because the study was considered to be a non-research public health activity. Informed written consent was obtained from all participants and from the guardians of minors.

## Results and discussions

### Data exploration

**Fig 1:**
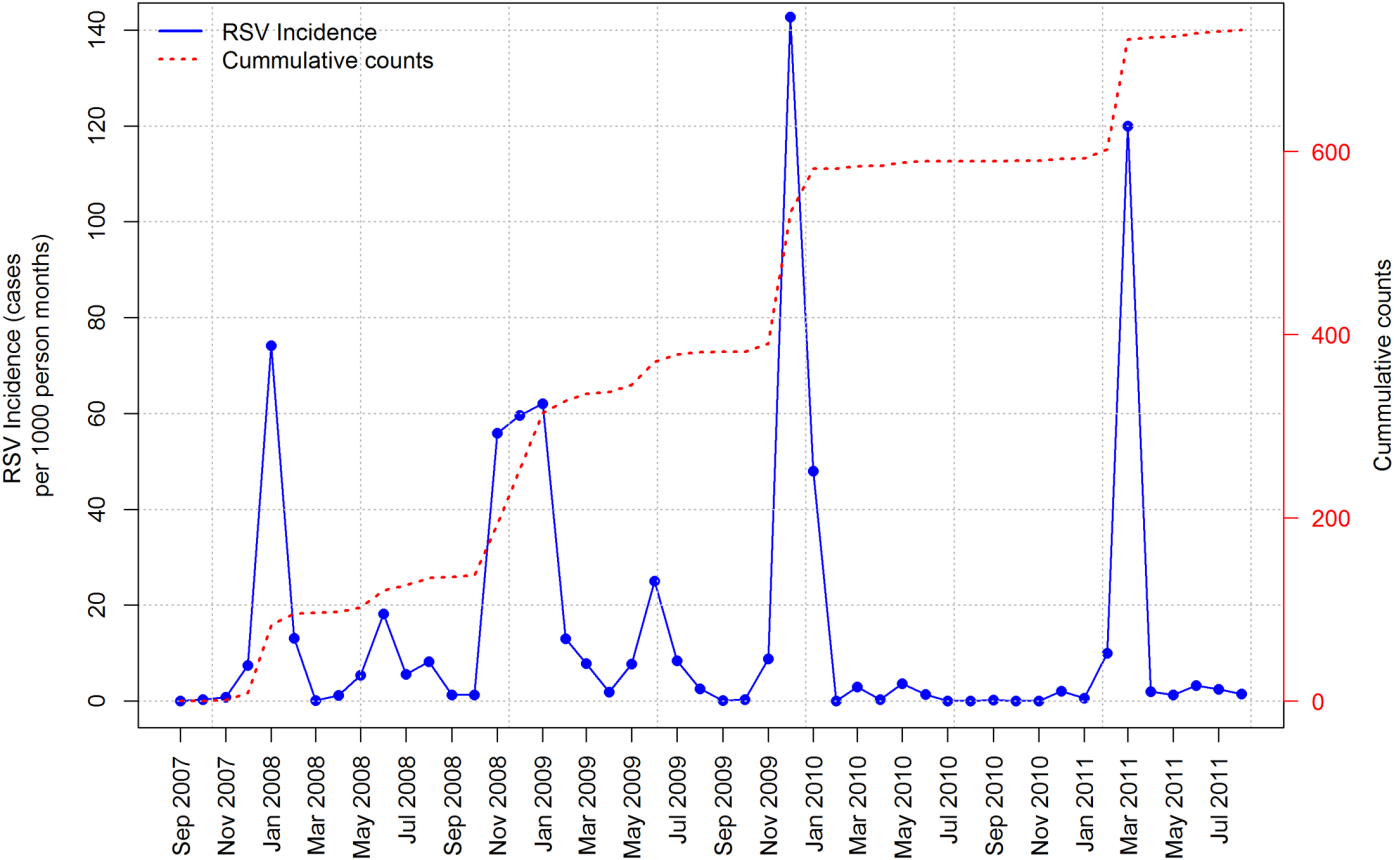
Plot of RSV incidence in Dadaab. Fluctuations in the data are roughly constant over time, indicating that the RSV time series could likely be described using an additive model.

A peak in RSV incidence occurred every 11–12 months, particularly from October to January (Fig 1). Other than these peaks, there was relatively low RSV incidence (≤ 20 cases per 1000 person months).

**Fig 2:**
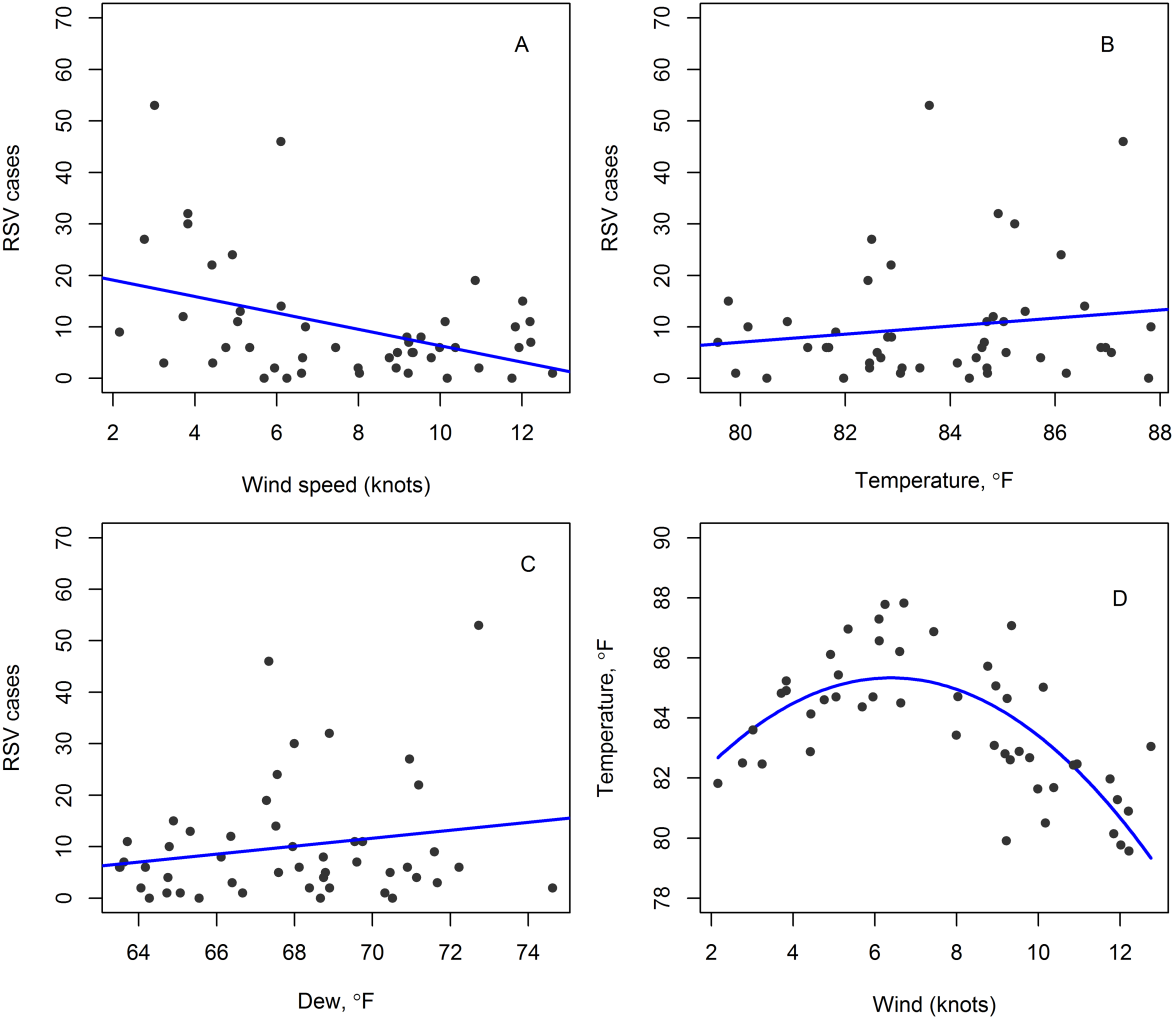
Correlation-regression analysis. A: Correlation between RSV incidence and wind speed; B: Correlation between RSV incidence and temperature; C: Correlation between RSV incidence and dew point; and D: Correlation between temperature and wind speed. In these plots, the regression lines of best fit are indicated by bold blue lines.

The decomposed data, seasonal pattern, trend line, and random component of the RSV, wind, rainfall, and temperature time series are shown in S1–S4 Figs. The seasonal pattern of RSV incidence regularly repeated itself, with two distinct peaks annually (S1 Fig). The data show that overall, lower wind speeds and higher temperatures were associated with higher RSV incidence. The magnitude of the seasonal components of the decomposed covariates did not vary annually (S1–S6 Figs). This justifies the use of additive, rather than multiplicative decomposition. There was a positive correlation between temperature and RSV incidence (Fig 2B). There was a significant moderate correlation between RSV incidence and wind speed (*ρ* = −1.603, *p* = 0.003)(Fig 2A); an insignificant weak correlation between RSV incidence and temperature(*ρ* = 0.809, *p* = 0.289) (Fig 2B); an insignificant weak correlation between RSV incidence and dew point (*ρ* = −0.763, *p* = 0.201) (Fig 2A); and for temperature and wind speed(Fig 2D);, the parabolic curve was fitted using:*x*_3_ = ɤ_0_ + ɤ_1_(*x*_1_−ɤ_2_)^2^ where ɤ_0,1,2_ are constants, and the regression fit was significant (*p* < 0.001). Here, *x*_1_ and *x*_3_ represent wind speed and temperature, respectively.

### Model assessment and comparison

**Fig 3:**
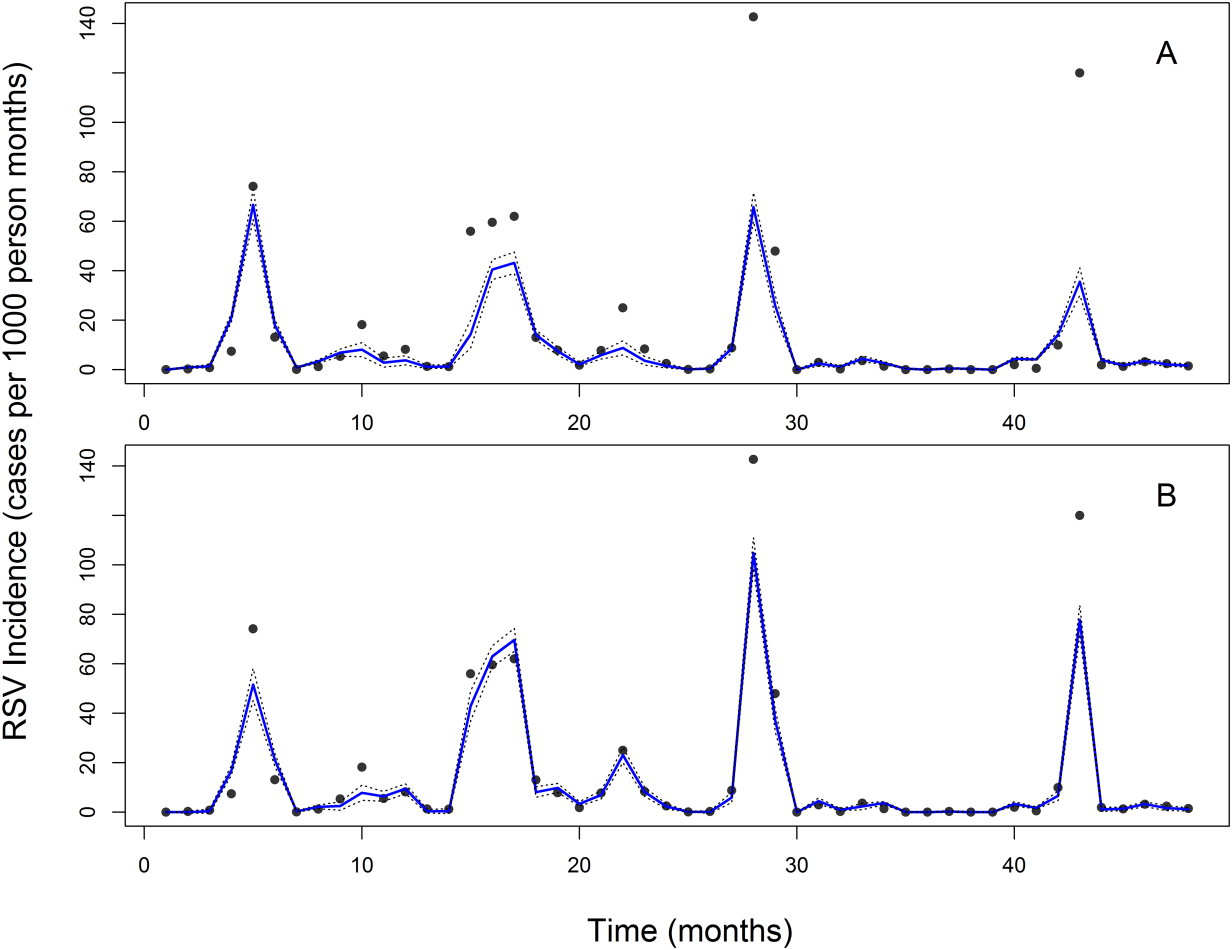
Best model fit to the RSV incidence data (bold lines) with decomposed covariates. A: Poisson, GLM. B: Poisson, GAM. The standard error bars to the model fit are indicated by the dotted lines (95% confidence bounds). The base year in all these plots was September 2009.

The trend component of the wind decomposition model decreased slightly immediately after 2008, then increased steadily to a peak in early 2009, followed by a decrease to a minimum value in late 2010 (S2 Fig). These finding indicate that the wind variable has a seasonal impact on RSV incidence. A similar seasonal effect was observed in the RSV and temperature decomposition (S1–S4 Figs). To determine the best predictive model, we compared the performance of the four models described in the methods section. The best GLMs and GAMs from the Poisson were compared using the AIC and residual deviances (S1 Table). In the models with decomposed covariates for both GLM and GAM, the current cases of RSV did not depend on the previous observations. The AIC was used to judge the best model from the set of models that had a good fit. The best models all had covariates with *p* < 0.05. This was the case for models with and without decomposed covariates. Of all the models that were evaluated, the Poisson GAM with decomposed covariates had the best fit to the data (AIC = 317.17 and a Deviance explained = 65.3%, S1 Table). Fig 3 shows the best model fit to the RSV incidence data with decomposed covariates comparing the Poisson GLM and the Poisson GAM, where the Poisson GAM fits the data well. The best model in its reduced form is the Poisson GAM^a^ (S4 Table). S2 Table contains the corresponding ANOVA results for the Poisson GAM^a^. From this table, the wind with both the trend and seasonal effects (seasonal effect of rainfall, trend mean dew point, and the trend effect of visibility) significantly explained RSV incidence. We note that time in months did not significantly explain RSV incidence, further demonstrating the importance of using climactic factors to explain the seasonality of RSV.

**Fig 4:**
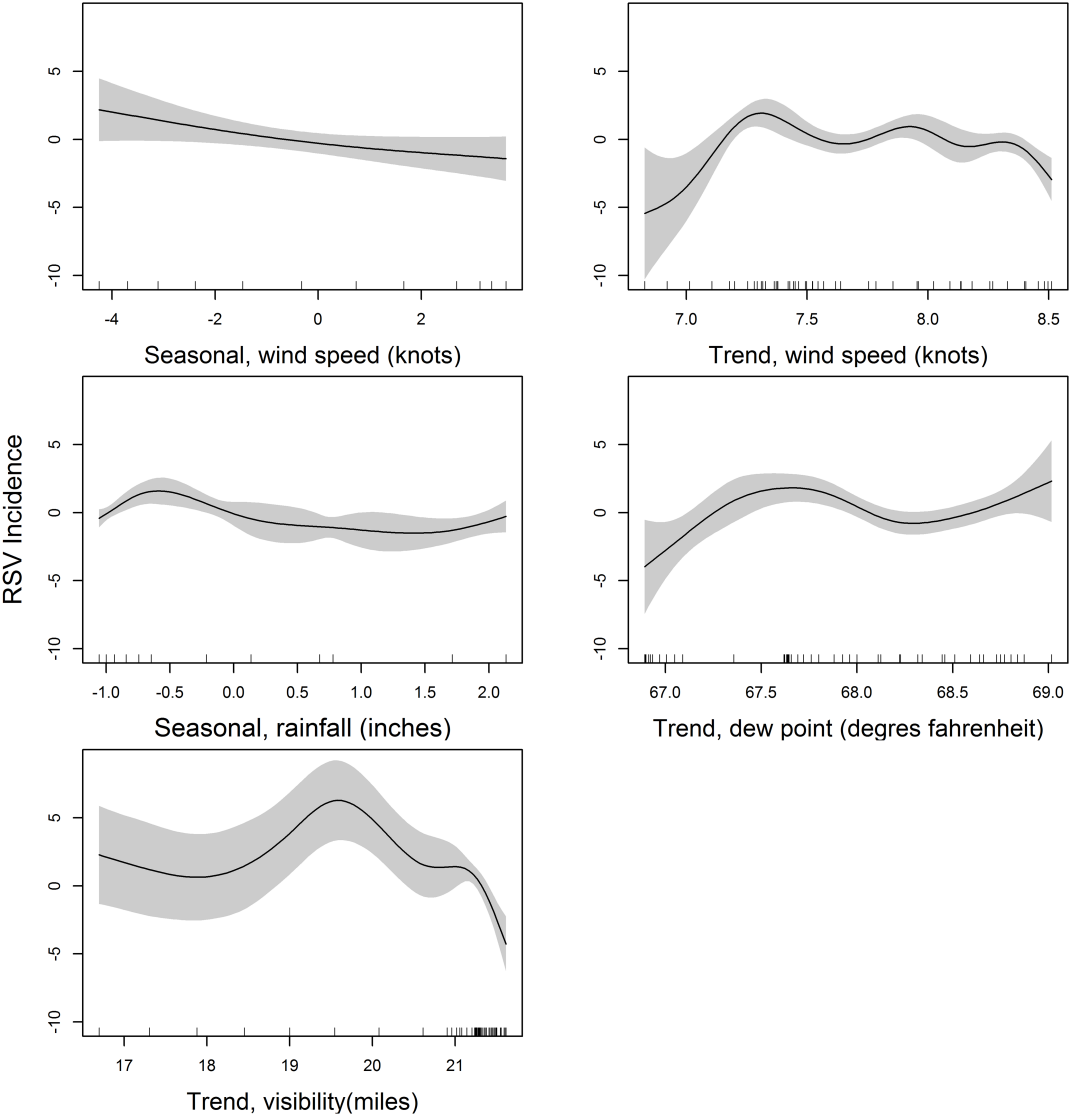
Best model fit (Poisson, GAM) to the RSV incidence data with the signifficant decomposed covariates. Seasonal, wind speed; Trend, wind speed; Seasonal, rainfall; Trend, rainfall; and Trend, visibility. The standard error bars to the model fit are indicated by the gray shade (95% confidence bounds). RSV incidence units as cases per 1,000 person months.

The direction of effects demonstrated nonlinear relationships with RSV incidence, except in the case of seasonal wind speed, which had a linear relationship (Fig 4). High wind speed within the same month had a significant negative effect on the RSV incidence. The trend component of the wind speed in the 2 months preceding incident RSV cases had a nonlinear relationship with RSV incidence. As the wind speed increased, incidence fluctuated from low to high, returning to low incidence when the speeds were highest. An increase in the seasonal component of rainfall in the four months preceding RSV cases was associated with an increase in RSV incidence. When rainfall was at its lowest, RSV incidence increased then returned to baseline when rainfall reached its maximum. The trend effect of the mean dew point 1 month preceding incident cases was associated with an increase in RSV incidence until dew point reached its maximum. The increase in visibility trend component 2 months preceding incident RSV cases demonstrated a constant effect on RSV incidence, which peaked when the visibility was 19.5 miles and troughed when the visibility was at its highest.

### Implication of results and comparison to related studies

Our data showed seasonal variations for RSV incidence (S1 Fig). The Poisson GAM with decomposed covariates out-performed the GLM variant, thereby relaxing its linearity. Generally, the role of climatic factors in determining disease dynamics is rather complex to decipher [32]. In the literature, there is strong evidence that the relationship between climatic factors and RSV incidence varies widely between geographical regions [18]. Previous studies have shown that climatic factors might be associated with RSV, although it remains unclear what these factors are or exactly how they impact RSV incidence. We performed a correlation analysis for each covariate with RSV by fitting regression lines to test the level of significance between the climatic variables (Fig 2A–2C). A recent study by Agoti et al [33] on RSV strains using the same RSV surveillance data showed that there were six epidemic peaks within the 3 year study period: two peaks each year; the first and the last peaks were composed of group B strains and the other four peaks were composed of group A strains. Agoti’s study, in conjunction with our findings, show that onset of RSV infections in Kenya can be reliably predicted. Our findings, in comparison with other studies, also suggest that the relationship between RSV incidence and climatic factors varies widely; for instance, from 2004 to 2012 in tropical and sub-tropical zones such as Hong Kong, China, Singapore, Kuala Lumpur, Malaysia, Medellin and Colombia outbreaks occurred primarily during the hot and rainy seasons [14].

The ability to predict increases in RSV incidence, based on prevailing meteorological conditions, could potentially inform the application of public health interventions and provisions of healthcare in Kenya, and perhaps, in other regions with a similar climate and equatorial location. Currently, there is no RSV vaccine available; however, in developed countries, infants at risk of severe outcomes can be administered monthly doses of the anti-RSV antibody, palivizumab, during outbreaks of RSV [3][8]. Because predicting the incidence of RSV could optimize the cost-effectiveness of immunoprophylaxis; our model might be useful to apply in a cost-benefit analysis of this approach in Kenya. In most temperate climate regions, RSV occurs as an annual epidemic. For instance, Noyola and Mandeville found that temperature was the predominant atmospheric condition explaining the annual spread and variability of RSV incidence in San Luis Potosi, Mexico [34]. Using correlation and regression analysis, Noyola and colleagues observed that the weekly number of RSV incidence between October 2002 and May 2006 was correlated to ambient temperature, barometric pressure, relative humidity, vapor tension, dew point, precipitation, and hours. Our findings corroborate what they observed for the same climatic factors. The modeling has aided identification of factors influencing RSV incidence and provided indicators for devising measures to prevent the spread of the disease.

Our analysis showed that other climatic factors affecting RSV seasonality can improve the performance of a predictive model. Khor et al [18] demonstrated that, in Malaysia, ambient temperature was inversely associated with RSV activity, even though the highest number of cases may not always coincide with the lowest temperature. A negative correlation between the mean minimum temperatures and RSV incidence was recently reported in Italy [11]. RSV transmission that occurs during cold weather is facilitated by its stability in secretions, since inhalation of cold air slows down the mucociliary escalator. This reduces phagocytic activity of leukocytes, increasing the host’s vulnerability to infection. There is evidence of RSV epidemics occurring in tropical areas with high temperatures during rainy seasons, a phenomenon that our data are exhibiting [35][36]. However, the exact mechanisms of how climatic factors affect RSV incidence requires further investigations, especially across geographically diverse regions. The relationship between the dynamics in wind speed and direction, and how these dynamics influence the climate of geographical regions like Dadaab, remains unclear. Understanding such complex relationships between the co-factors explaining the spread of RSV is essential to predict its incidence.

A foreseeable limitation of our models is that with log- or logit-links; the mean value zero corresponds to an infinite range on a linear predictor scale. For count data with a relatively large number of zeros clustered closely within the covariate space, GAMs might suffer from identifiability problems, especially the Poisson family. For the over-dispersion parameter, the assumption of equal mean and variance inherent in the Poisson GAM might be violated; hence, it has to be replaced by variances that exceed the mean. Our data show a cyclic and seasonal behavior for RSV incidence among children (Fig 1). The Poisson GAM from this analysis demonstrated that climatic factors, including wind speed, rainfall, dew point and visibility, significantly affected RSV incidence. The use of atmospheric condition data help public health officials predict increases in RSV infection incidence among children and help them prepare and respond more swiftly to increasing RSV incidence in low-resource regions or communities. While specific vaccines, antiviral medications, and immunoglobulins are not available to control RSV in these settings, agencies responsible for managing healthcare in crisis-affected populations can increase preparedness for RSV outbreaks by establishing additional patient-isolation areas and bed space, ensuring that all healthcare workers are provided with adequate personal protective equipment (e.g., facial masks and gloves) and appropriate amounts of hand sanitizers and adequate hand-washing facilities for healthcare workers are readily available.

Health education is important; crisis-affected populations should be made aware of the symptoms and signs of RSV, how it spreads, and how to protect themselves and their loved ones. Health education should focus on how to cover coughs, keep appropriate social distancing (e.g., not being too close to others, not shaking hands), and the importance of washing hands with soap. In particular, our model indicates that when the wind speed in knots change from high to low, these interventions should be enhanced to prevent spread of RSV infections in Kenya. In the future, these models could be validated with new RSV surveillance data to see how well they perform to predict increases in RSV incidence particularly for geographical regions with similar climatic attributes to Dadaab.

## Supporting information

S1 Fig Decomposition of RSV time-series data. The variation in the remainder component is approximately the same as the variation in the data. The variation in the seasonal and trend components are about 3–4x smaller than that observed in the data. The long-term trend components appear to be generally increasing. The random (remainder), the bottom plot, show the residual variation in the data after the long-term trend and seasonality are removed.
(TIFF)

S2 Fig Decomposition of wind time-series data. The variation in the trend is much smaller than that in the data. The variations in the seasonal and remainder components are marginally smaller than the variation in the data (gray bars on the right).
(TIFF)

S3 Fig Decomposition of rainfall time-series data. The variations in the seasonal and remainder components do not deviate much from that in the data. The variation in the trend component is roughly 4x less than the variation in the data.
(TIFF)

S4 Fig Decomposition of temperature time-series data. The trend component has a much smaller variation than that in the data. The seasonal and remainder components show marginally smaller variation than that observed in the data. The long-term trend components appear to be generally increasing.
(TIFF)

S5 Fig Decomposition of dew time-series data. The trend exibits approximately 3x the overall variation in the Dew data (large gray bar relative to the gray bar on the right-hand of the data plot). The long-term trend components appear to be generally increasing.
(TIFF)

S6 Fig Decomposition of visibility time-series data. The season accounts for a very small portion of the overall variation in the visibility value (large gray bar relative to the gray bar on the right-hand of the data plot). The long-term trend components seem to be generally decreasing.
(TIFF)

S1 Table Model diagnostic and performance results. (DOCX)

S2 Table ANOVA model for the best performing model, the Poisson GAM with covariate decomposition. (DOCX)

S3 Table Covariates and their description. Non-decomposed and decomposed covariates into the seasonal (S), trend (T), and random (R) components.
(DOCX)

S4 Table Selected Poisson candidate models. (DOCX)
