# Supplementary figures and images for: Effect of climate on incidence of respiratory syncytial virus infections in a refugee camp in Kenya: A non-Gaussian time-series analysis
Source: PLoS One. 2017 Jun 1;12(6):e0178323. doi: 10.1371/journal.pone.0178323 (PMC5453485; doi:10.1371/journal.pone.0178323)

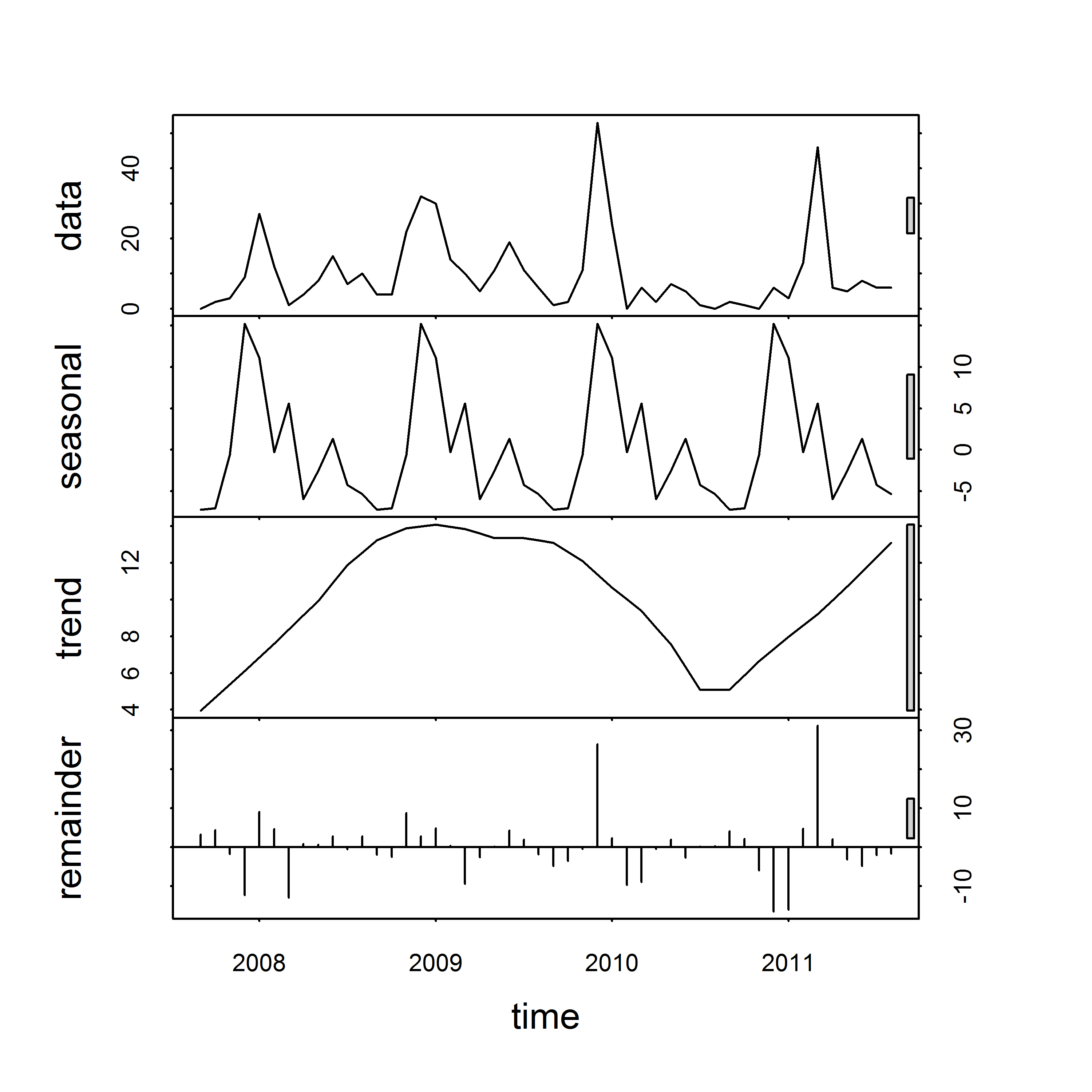

Supplement: S1 Fig — The variation in the remainder component is approximately the same as the variation in the data. The variation in the seasonal and trend components are about 3–4x smaller than that observed in the data. The long-term trend components appear to be generally increasing. The random (remainder), the bottom plot, show the residual variation in the data after the long-term trend and seasonality are removed. (TIFF) [file pone.0178323.s001.tiff]

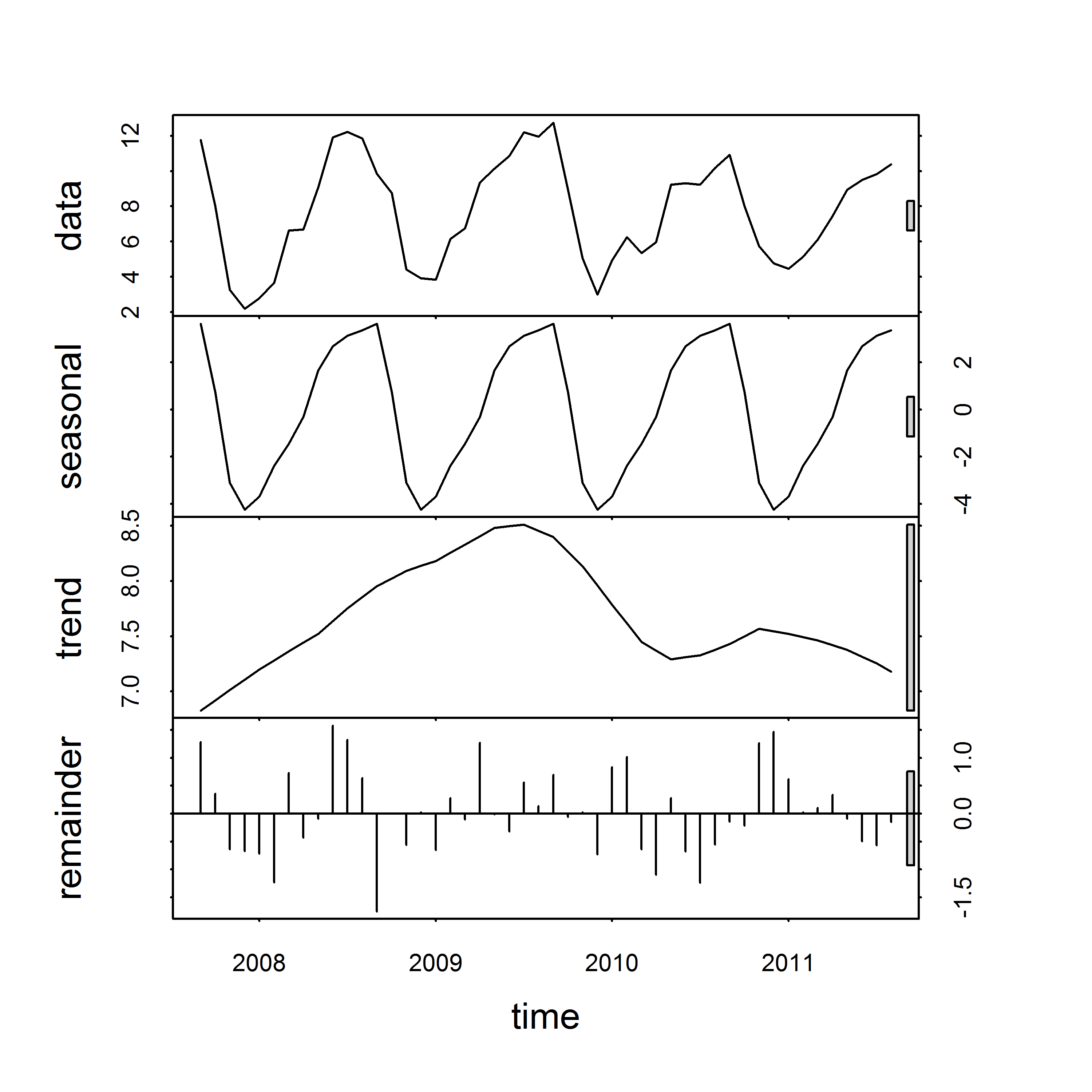

Supplement: S2 Fig — The variation in the trend is much smaller than that in the data. The variations in the seasonal and remainder components are marginally smaller than the variation in the data (gray bars on the right). (TIFF) [file pone.0178323.s002.tiff]

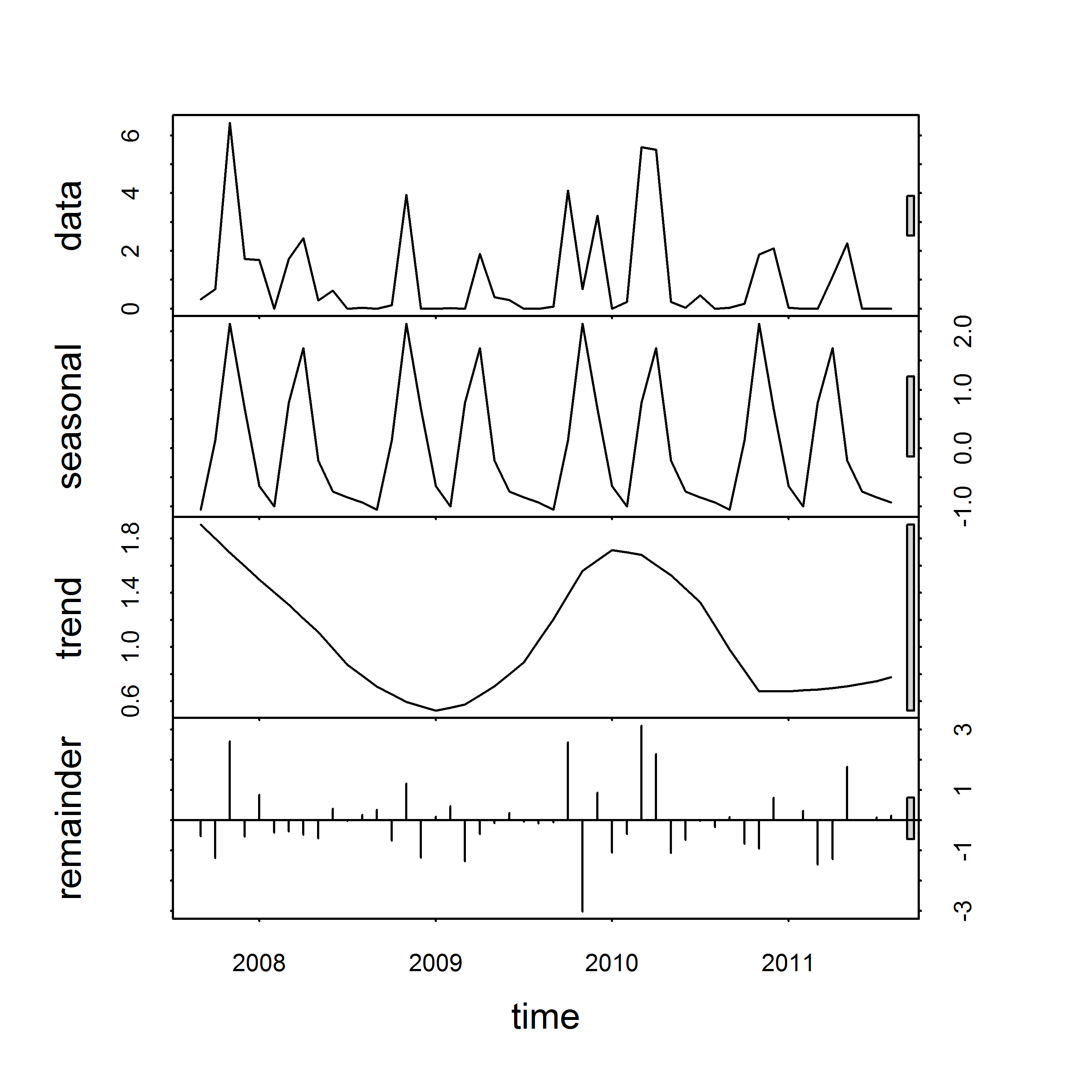

Supplement: S3 Fig — The variations in the seasonal and remainder components do not deviate much from that in the data. The variation in the trend component is roughly 4x less than the variation in the data. (TIFF) [file pone.0178323.s003.tiff]

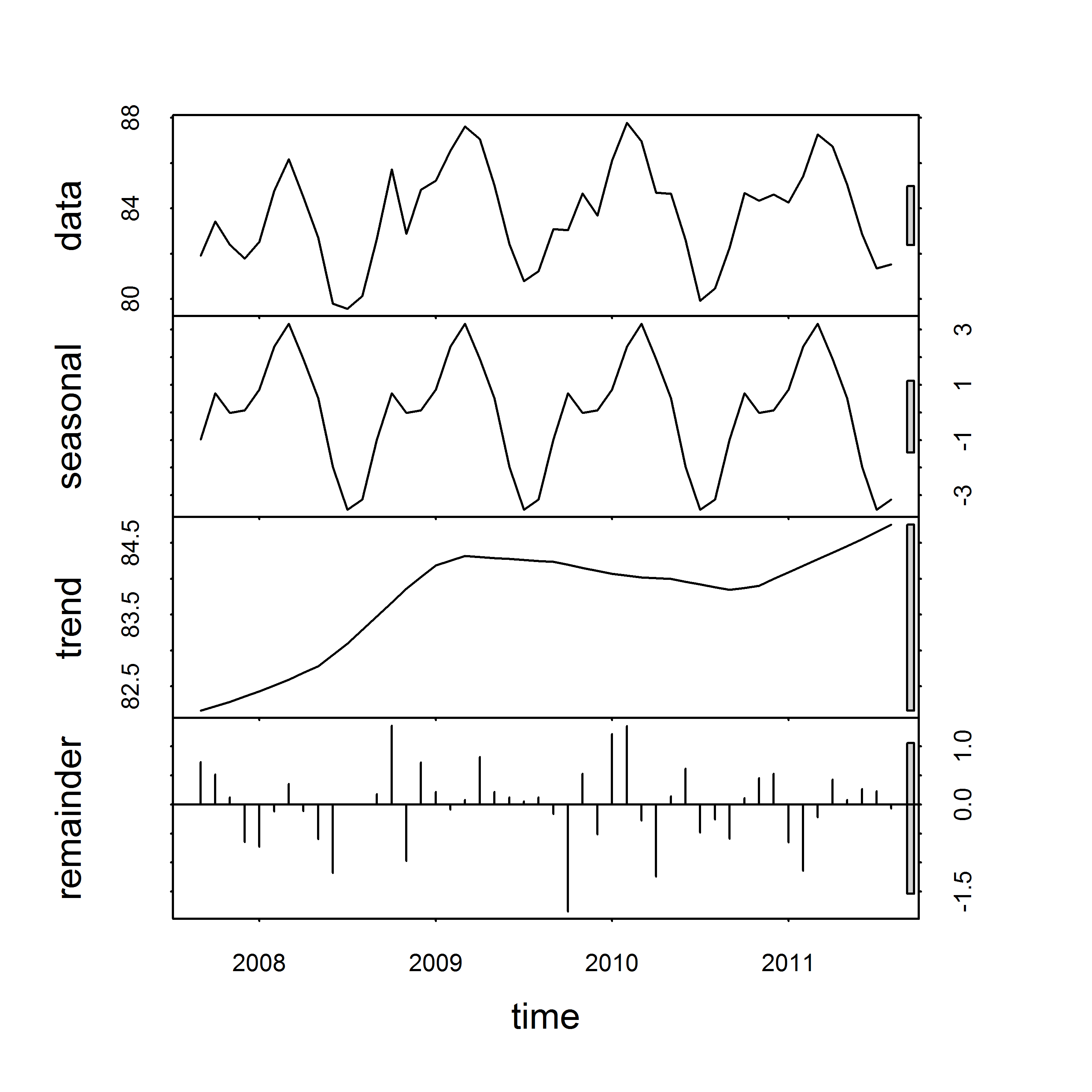

Supplement: S4 Fig — The trend component has a much smaller variation than that in the data. The seasonal and remainder components show marginally smaller variation than that observed in the data. The long-term trend components appear to be generally increasing. (TIFF) [file pone.0178323.s004.tiff]

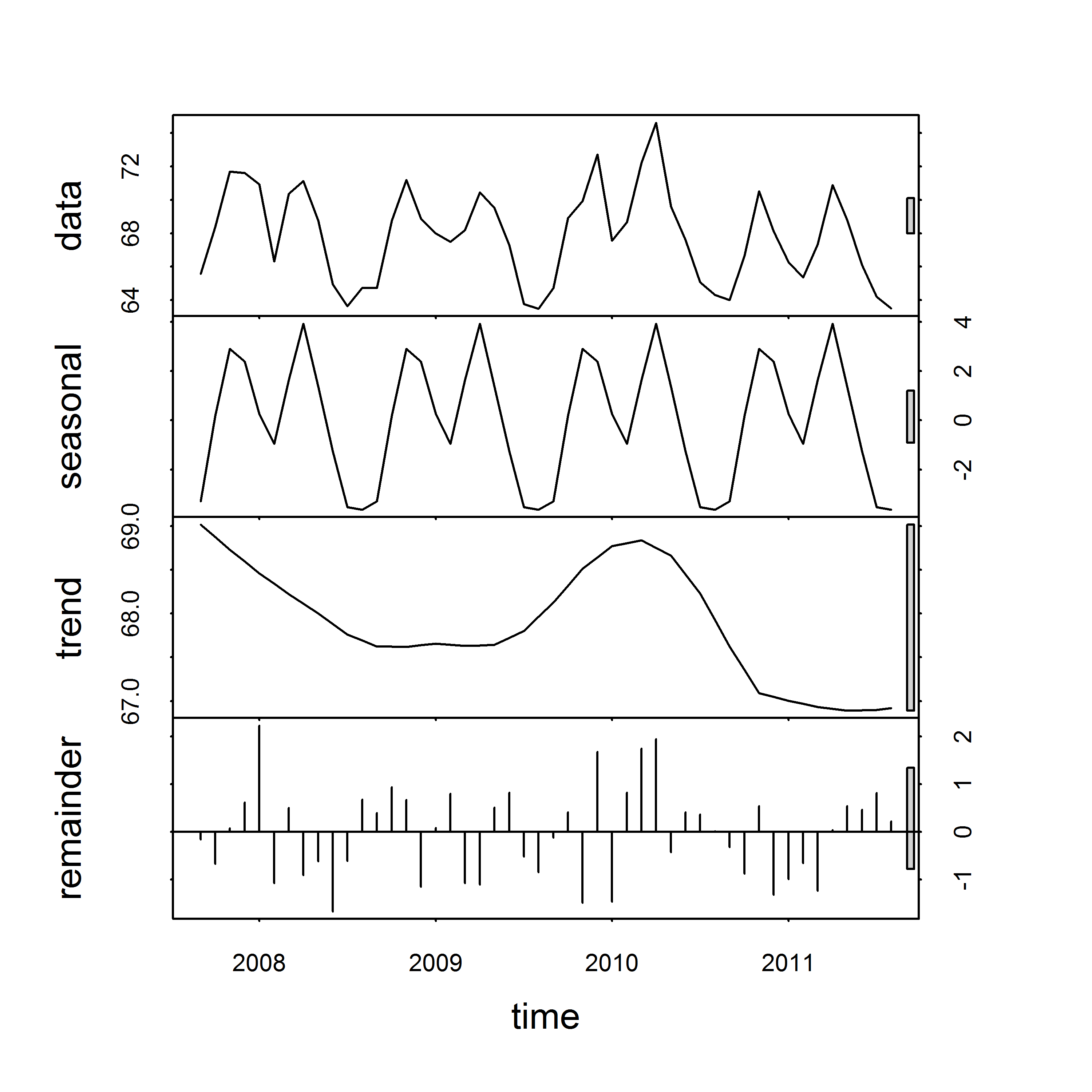

Supplement: S5 Fig — The trend exibits approximately 3x the overall variation in the Dew data (large gray bar relative to the gray bar on the right-hand of the data plot). The long-term trend components appear to be generally increasing. (TIFF) [file pone.0178323.s005.tiff]

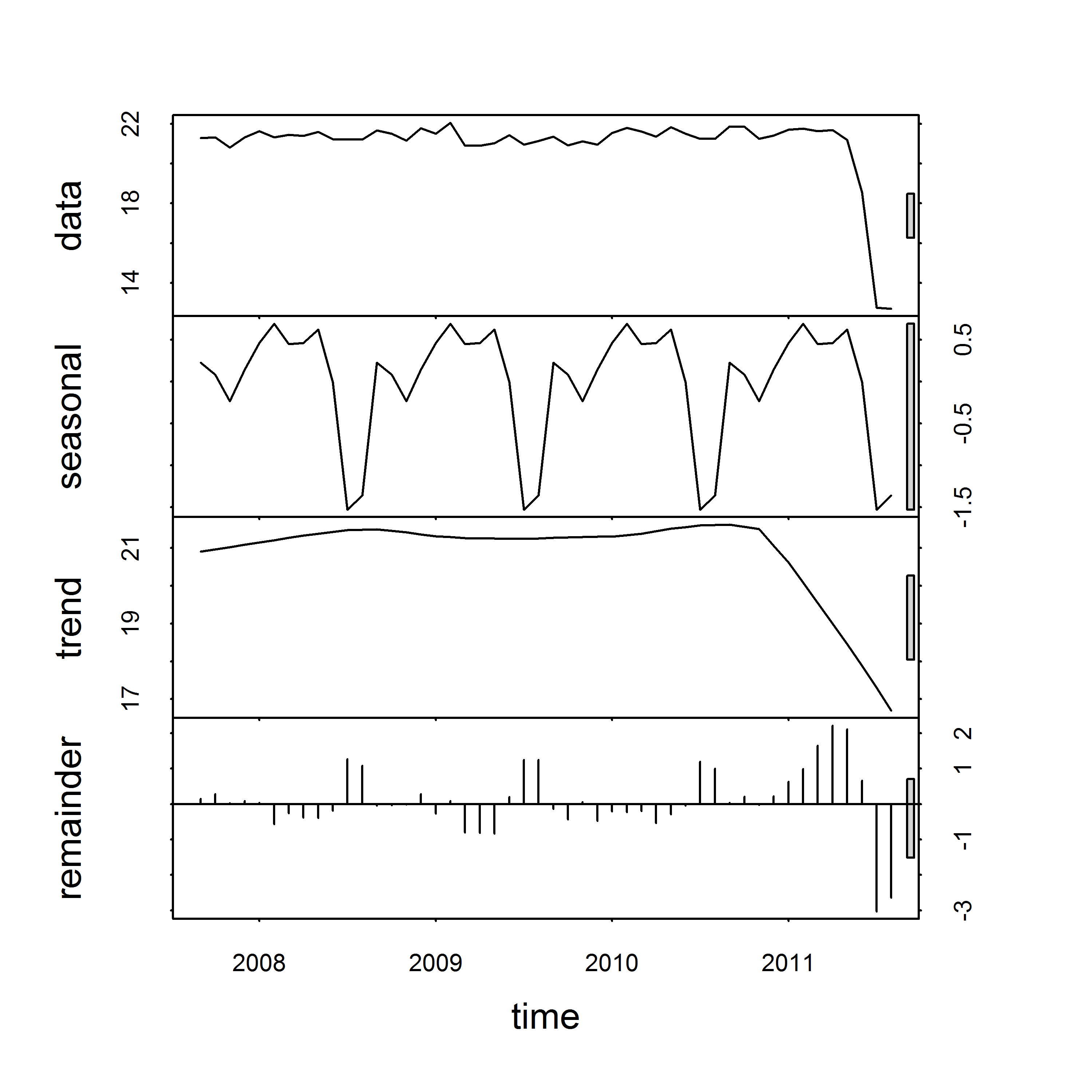

Supplement: S6 Fig — The season accounts for a very small portion of the overall variation in the visibility value (large gray bar relative to the gray bar on the right-hand of the data plot). The long-term trend components seem to be generally decreasing. (TIFF) [file pone.0178323.s006.tiff]
