# Supplementary material for: Effect of climate on incidence of respiratory syncytial virus infections in a refugee camp in Kenya: A non-Gaussian time-series analysis
Source: PLoS One. 2017 Jun 1;12(6):e0178323. doi: 10.1371/journal.pone.0178323 (PMC5453485; doi:10.1371/journal.pone.0178323)
Supplement: S1 Table — (DOCX) [file pone.0178323.s007.docx]

| Model | Deviance explained (%) | | AIC |
| --- | --- | --- | --- |
| Poisson GLM^a^ | 34.3 | 446.86 | |
| Poisson GLM^b^ | 29.4 | 477.25 | |
| Poisson GAM^a^ | 65.3 | 317.17 | |
| Poisson GAM^b^ | 59.5 | 346.44 | |

The superscripts ^a,b^ indicate models with and without covariate decomposition, respectively.
