# Supplementary material for: Effect of climate on incidence of respiratory syncytial virus infections in a refugee camp in Kenya: A non-Gaussian time-series analysis
Source: PLoS One. 2017 Jun 1;12(6):e0178323. doi: 10.1371/journal.pone.0178323 (PMC5453485; doi:10.1371/journal.pone.0178323)
Supplement: S2 Table — (DOCX) [file pone.0178323.s008.docx]

| Variable | lag | df | $F$-value | $p$-value |
| --- | --- | --- | --- | --- |
| Seasonal, wind speed | 1 | 4 | 28.81 | <0.0001 |
| Trend, wind speed | 3 | 4 | 17.99 | 0.0012 |
| Seasonal, rainfall | 0 | 4 | 27.70 | <0.0001 |
| Trend, mean dew point | 2 | 4 | 45.59 | <0.0001 |
| Trend, visibility | 2 | 4 | 68.18 | <0.0001 |
| Month |  | 3 | 2.48 | 0.4784 |
