# Supplementary material for: Effect of climate on incidence of respiratory syncytial virus infections in a refugee camp in Kenya: A non-Gaussian time-series analysis
Source: PLoS One. 2017 Jun 1;12(6):e0178323. doi: 10.1371/journal.pone.0178323 (PMC5453485; doi:10.1371/journal.pone.0178323)
Supplement: S3 Table — Non-decomposed and decomposed covariates into the seasonal (S), trend (T), and random (R) components. (DOCX) [file pone.0178323.s009.docx]

| **Covariate** | **Description** |
| --- | --- |
| *x*_t1_ | Wind speed |
| *x*_t2_ | amount of Rainfall |
| *x*_t3_ | Temperatures |
| *x*_t4_ | mean Dew point |
| *x*_t5_ | Visibility |
| *x*_t1S_ | Seasonal, wind |
| *x*_t1T_ | Trend, wind |
| ξ_t1R_ | Random, wind |
| *x*_t2S_ | Seasonal, rainfall |
| x_t2T_ | Trend, rainfall |
| ξ_t2R_ | Random, rainfall |
| *x*_t3S_ | Seasonal, temperature |
| *x*_t3T_ | Trend, temperature |
| ξ_t3R_ | Random, temperature |
| *x*_t4S_ | Seasonal, dew |
| x_t4T_ | Trend, dew |
| ξ_t4R_ | Random, dew |
| *x*_t5S_ | Seasonal, visibility |
| x_t5T_ | Trend, visibility |
| ξ_t5R_ | Random, visibility |
