# Supplementary material for: Effect of climate on incidence of respiratory syncytial virus infections in a refugee camp in Kenya: A non-Gaussian time-series analysis
Source: PLoS One. 2017 Jun 1;12(6):e0178323. doi: 10.1371/journal.pone.0178323 (PMC5453485; doi:10.1371/journal.pone.0178323)
Supplement: S4 Table — (DOCX) [file pone.0178323.s010.docx]

| **Covariates** | **GLM^a^** | **GLM^b^** | **GAM^a^** | **GAM^b^** |
| --- | --- | --- | --- | --- |
|  | **Est(se)** | **Est(se)** | **Est(se)** | **Est(se)** |
| Intercept | -0.69(0.074)*** | -0.71(0.070)*** | -2.71(4.111) | -2.09(0.637)** |
| *y_t-1_* | 0.004(0.004) | 0.02(0.004) *** | -0.0006(0.007) | 0.01 (0.006). |
| *x*_t1_ |  | -0.82(0.441). |  |  |
| *x*_t2_ |  | -1.21(0.427) ** |  |  |
| *x*_t3_ |  | -2.43(0.408) *** |  |  |
| *x*_t4_ |  | 1.33(0.454) ** |  |  |
| *x*_t1S_ | -6.45(0.917) *** |  |  |  |
| x_t1T_ | 1.69(0.286) *** |  |  |  |
| *x*_t2S_ | -1.12(0.246) *** |  |  |  |
| *x*_t4T_ | 0.630(0.254) * |  |  |  |
| x_t5T_ | -1.22(0.423) ** |  |  |  |
| Cos(2πt/12) | 1.27(0. 277)*** | -0.70(0.182)*** |  |  |
| Sin(2πt/12) | -1.20(0.210) *** |  |  |  |
| ns(x_t1_,4)1 |  |  |  | -5.45(0.792)*** |
| ns(x_t1_,4)2 |  |  |  | -1.67 (0.784)* |
| ns(x_t1_,4)3 |  |  |  | -0.32 (1.287) |
| ns(x_t1_,4)4 |  |  |  | -1.78(0.723)* |
| ns(x_t2_,4)1 |  |  |  | -1.03(0.337)** |
| ns(x_t2_,4)2 |  |  |  | 1.51(0.399)*** |
| ns(x_t2_,4)3 |  |  |  | 0.00(0.000) |
| ns(x_t2_,4)4 |  |  |  | -3.37(0.614)*** |
| ns(x_t3_,4)1 |  |  |  | -1.41(0.484)** |
| ns(x_t3_,4)2 |  |  |  | -1.34(0.602)* |
| ns(x_t3_,4)3 |  |  |  | -1.25 (1.043) |
| ns(x_t3_,4)4 |  |  |  | -0.14(0.687) |
| ns(x_t4_,4)1 |  |  |  | 2.80(0.448)*** |
| ns(x_t4_,4)2 |  |  |  | 2.43(0.490)*** |
| ns(x_t4_,4)3 |  |  |  | 4.72(0.997)*** |
| ns(x_t4_,4)4 |  |  |  | 3.21(0.732)*** |
| ns(x_t1S_, 4)1 |  |  | -10.97(6.153). |  |
| ns(x_t1S_, 4)2 |  |  | -3.71(2.241). |  |
| ns(x_t1S_, 4)3 |  |  | -13.25(10.110) |  |
| ns(x_t1S_, 4)4 |  |  | -7.19(4.857) |  |
| ns(x_t1T_, 4)1 |  |  | 2.86(1.406)* |  |
| ns(x_t1T_, 4)2 |  |  | -0.07(1.076) |  |
| ns(x_t1T_, 4)3 |  |  | 1.01(2.711) |  |
| ns(x_t1T_, 4)4 |  |  | 2.05(0.700)** |  |
| ns(x_t2S_, 4)1 |  |  | -2.31(3.019) |  |
| ns(x_t2S_, 4)2 |  |  | 3.87(2.245). |  |
| ns(x_t2S_, 4)3 |  |  | -0.88(2.411) |  |
| ns(x_t2S_, 4)4 |  |  | 0.46(0.781) |  |
| ns(x_t4T_, 4)1 |  |  | 4.12(1.307)** |  |
| ns(x_t4T_, 4)2 |  |  | 7.33(1.125)*** |  |
| ns(x_t4T_, 4)3 |  |  | 13.36(2.437)*** |  |
| ns(x_t4T_, 4)4 |  |  | -2.032(0.948)* |  |
| ns(x_t5T_, 4)1 |  |  | -6.31(1.646)*** |  |
| ns(x_t5T_, 4)2 |  |  | -5.53(1.563)*** |  |
| ns(x_t5T_, 4)3 |  |  | -3.17(1.835). |  |
| ns(x_t5T_, 4)4 |  |  | -10.03(1.334)*** |  |
| ns(t, 4)1 |  |  | 5.65(4.180) | 2.08(1.156) . |
| ns(t, 4)2 |  |  | 5.87(4.794) | 1.01(0.786) |
| ns(t, 4)3 |  |  | 0.00(0.000 | 2.49(0.666) *** |
| ns(t, 4)4 |  |  | -5.57 (4.299) | -1.43(0.326) *** |

Signif. codes: $p<0$ ‘***’; $p<0.001$ ‘**’; $p<0.01$ ‘*’; $p<0.05$ ‘.’; $p<0.1$ ‘ ’; $p<1$

ns stands for natural splines, and the numbers inside and outside the brackets represent the degres of freedom for the splines.
